# Association of the Protein-Quality-Control Protein Ubiquilin-1 With Alzheimer’s Disease Both *in vitro* and *in vivo*

**DOI:** 10.3389/fnins.2022.821059

**Published:** 2022-03-17

**Authors:** Can Zhang, Shivangi M. Inamdar, Swathi Swaminathan, Daniel R. Marenda, Aleister J. Saunders

**Affiliations:** ^1^Department of Biology, Drexel University, Philadelphia, PA, United States; ^2^Division of Biological Infrastructure, National Science Foundation, Alexandria, VA, United States

**Keywords:** Alzheimer’s disease, ubiquilin 1 (UBQLN1), *Drosophila*, APP – amyloid precursor protein, gamma secretase (γ-secretase)

## Abstract

Alzheimer’s disease (AD) belongs to a class of diseases characterized by progressive accumulation and aggregation of pathogenic proteins, particularly Aβ proteins. Genetic analysis has identified *UBQLN1* as an AD candidate gene. Ubiquilin-1 levels reduce with AD progression, suggesting a potential loss-of-function mechanism. The ubiquilin-1 protein is involved in protein quality control (PQC), which plays essential roles in cellular growth and normal cell function. Ubiquilin-1 regulates γ-secretase by increasing endoproteolysis of PS1, a key γ-secretase component. Presently, the effects of ubiquilin-1 on cellular physiology as well as Aβ-related events require further investigation. Here, we investigated the effects of ubiquilin-1 on cellular growth and viability in association with APP (amyloid-β protein precursor), APP processing-related β-secretase (BACE1, BACE) and γ-secretase using cell and animal-based models. We showed that loss-of-function in *Drosophila ubqn* suppresses human APP and human BACE phenotypes in wing veins and altered cell number and tissue compartment size in the wing. Additionally, we performed cell-based studies and showed that silencing *UBQLN1* reduced cell viability and increased caspase-3 activity. Overexpression of *UBQLN1* significantly reduced Aβ levels. Furthermore, pharmacological inhibition of γ-secretase increased ubiquilin-1 protein levels, suggesting a mechanism that regulates ubiquilin-1 levels which may associate with reduced Aβ reduction by inhibiting γ-secretase. Collectively, our results support not only a loss-of-function mechanism of ubiquilin-1 in association with AD, but also support the significance of targeting ubiquilin-1-mediated PQC as a potential therapeutic strategy for AD.

## Introduction

Alzheimer’s disease (AD) is pathologically characterized by accumulation of neuronal amyloid plaques and fibrillary tangles, which ultimately lead to neuronal death and dementia (Hardy and Selkoe, 2002; Choi et al., 2014). The main components of the amyloid plaques are beta-amyloid peptides (or Aβ), which are primarily 37–43 amino acids long and generated by the proteolytic processing of a type-I transmembrane glycoprotein, amyloid-β protein precursor (APP) (Tanzi et al., 1987; Tanzi and Bertram, 2001). APP is present on almost every membrane containing intracellular compartment. After translation, APP is transported onto the membrane surface through intracellular trafficking. Aβ generation, also known as amyloidogenesis, requires two proteolytic processing events of APP mediated by β-secretase (BACE) and γ-secretase. Like APP, BACE is also a type-I transmembrane protein, while γ-secretase is a membrane protein complex which contains presenilin1 (PS1), nicastrin (NCSTN), Aph-1, and Pen-2. γ-Secretase cleavage can yield different length of Aβ species, among which Aβ40 and Aβ42 are the two main components in AD brains. Aβ42 is more aggregation prone than Aβ40 and increased Aβ42/Aβ40 ratios are common in early onset familial AD (FAD) cases. APP can also be cleaved by α-secretase, which cleaves within the Aβ sequence and precludes the process of amyloidogenesis (Zhang and Saunders, 2007).

In the past decade, there has been increasing interest in identifying and characterizing novel candidate genes in association with the progression of AD. One such gene of interest implicated in AD is *UBQLN1*, which encodes ubiquilin-1, a ubiquitin-like protein. Polymorphism of *UBQLN1*, has been shown to increase the risk of AD in family-based and large case-control samples (Bertram et al., 2005; Bensemain et al., 2006; Kamboh et al., 2006). Biologically, ubiquilin-1 is an intracellular protein quality control (PQC) protein and regulates related protein functions at various organelles or systems, particularly the ubiquitin-proteasome system (UPS) (Funakoshi et al., 2002; Ko et al., 2002; Medicherla et al., 2004; El Ayadi et al., 2013). Pathologically, ubiquilin-1 has been implicated to be involved in regulation of molecular process of AD (Hiltunen et al., 2006; Li et al., 2007; Zhang et al., 2007; Ganguly et al., 2008; Gross et al., 2008; Adegoke et al., 2017). Furthermore, ubiquilin-1 is associated with non-AD disorders and conditions, including Parkinson’s disease and a variety of cancers (Mah et al., 2000; Wang et al., 2006; Chen et al., 2007). These findings suggest a broad and important role of ubiquilin-1 in human pathophysiology. As a PQC protein, ubiquilin-1 levels reduce with AD progression, suggesting a potential link to AD pathology by a loss-of-function mechanism (Stieren et al., 2011). Related to its function in PQC, ubiquilin-1 has also been shown as an APP chaperon protein, reduction of which may lead to overproduction of pathogenic APP cleavage fragments and neuronal death (Stieren et al., 2011). Furthermore, ubiquilin-1 regulates γ-secretase by affecting the endoproteolysis of γ-secretase-related PS1, which harbors proteolytic cleavage activity of γ-secretase (Mah et al., 2000; Hiltunen et al., 2006; Zhang et al., 2007). Studies have also shown that ubiquilin-1 directly affects BACE trafficking and localization to recycling endosomal compartments of neuronal cells, where Aβ generation is most abundant (Takalo et al., 2017). Currently, the effects of ubiquilin-1 on AD-related events as well as the interaction of BACE and APP-related mechanistic phenotypes require further investigation.

Here, we investigated the effects of ubiquilin-1 on PQC and association with APP processing-related β-secretase and γ-secretase using cell and animal-based models. We analyzed the function of the *Drosophila* homolog of ubiquilin-1 (CG14224, *ubqn*) in the developing wing. *Drosophila* serves as a suitable *in vivo* system to model AD-associated protein function, as the fruit fly contains functional homologs of all the necessary components of the γ-secretase complex (Guo et al., 2003; Ganguly et al., 2008; Chakraborty et al., 2011), and can process expressed human APP (Fossgreen et al., 1998), displaying adult phenotypes that are easily identifiable and have broad phenotypic ranges for genetic studies. Additionally, human β-secretase can be expressed in fly tissues, along with human APP (Greeve et al., 2004) to generate adult phenotypes. Following animal studies, we also characterized the effects of silencing *UBQLN1* on cell viability *in vitro* using the human neuroblastoma SH-SY5Y cells, as well as the effects of increasing *UBQLN1* in AD-related Aβ levels. Thus, the results of our cell and animal-based studies may collectively be useful to further elucidate the underlying mechanism of ubiquilin-1 as a PQC protein in association with AD.

## Materials and Methods

### *Drosophila* Culture and Genetics

All flies were raised on standard cornmeal/molasses/agar media at 25°C. Experimental genotypes were generated using the Gal4/UAS system for expressing specific proteins in a temporal and tissue specific manner, as previously described (Brand and Perrimon, 1993). All crosses were performed using *engrailed:Gal4* (Brower, 1986), which expresses the Gal4 protein specifically within the posterior compartment of *Drosophila* wings. The anterior compartment served as an internal control for each experiment. Transgenic *Drosophila* genotypes used were: *UAS:APP* (Fossgreen et al., 1998), *UAS:APP* and *UAS:BACE* (Greeve et al., 2004), *UAS:p35* (Bloomington Stock Center # 5072), *UAS:ubiquilin RNAi* (Vienna *Drosophila* RNAi Center Transformant ID#s 47448 and 47449). *UAS:GFP* (Bloomington Stock Center # 4775) was used as the wild type control.

### Fly Wing Analysis

All wings were dehydrated in ethanol and mounted in DPX (Zeiss). Digital photographs were taken under a Leica mZ 12.5 stereomicroscope using an attached Leica digital camera. Cell count and surface area measurements were performed as previously described (Marenda et al., 2003), using NIH Image J for analysis.

### Chemical, Antibodies, and Plasmids

L-685,458 and puromycin were purchased from Sigma. β-Actin antibody (1:10,000) was purchased from Sigma. The APP C-terminal antibody (A8717; 1:1,000) was purchased from Sigma. The ubiquilin-1 antibody (1:160) was purchased from Zymed. The HRP-conjugated secondary antibodies (anti-mouse and anti-rabbit) (1:10,000) were purchased from GE. The plasmids APP-Gal4, Gal4-UAS-luciferase (encoding firefly luciferase) were kindly provided from Dr. Thomas Südof, and have been described (Cao and Sudhof, 2001). The previously reported ubiquilin-1 expressing plasmid was constructed from the pCMV vector and was kindly provided by Dr. Mervyn J. Monteiro (Mah et al., 2000).

### Cell Culture

SH-SY5Y and HEK-293 cells were purchased from the American Type Culture Collection (ATCC). These cell lines were cultured in Dulbecco’s modified Eagle’s medium (DMEM) supplemented with 10% fetal bovine serum, 2 mM L-glutamine, 100 units/ml penicillin, and 100 μg/ml streptomycin. SH-SY5Y cells that stably express APP-Gal4 and the Gal4-UAS-luciferase reporter construct (named as “SY5Y-APP-Gal4 cells“) were described elsewhere (Zhang et al., 2007). These cells were maintained in media containing 200 μg/ml G418. SY5Y-APP-Gal4 cells were transfected with non-silencing or control shRNA construct (not targeting any known genes) or shUbqln1 construct (targeting ubiquilin-1 mRNA or “shUBQLN1”) (Zhang et al., 2007). The multiple clonal cells containing puromycin-resistant clones were selected and continued to grow in 1 μg/ml puromycin and 200 μg/ml G418-containing media. When cells reached 15 splitting times, they were replaced with fresh batches.

### 3-(4,5-Dimethylthiazol-2-yl)-2,5-Diphenyl-2H-Tetrazolium Bromide Assay

The 3-(4,5-dimethylthiazol-2-yl)-2,5-diphenyl-2H-tetrazolium bromide (MTT) assay was carried out using the MTT cell proliferation assay kit (ATCC Bioproducts™) according to the manufacturer’s instructions. Briefly, 5,000 cells were plated per well in the 96-well plates. Cells were challenged with 500 μM H_2_O_2_ for 1 h and 10 μl MTT reagent was added to the cells. After 3 h of incubation at 37°C, 100 μl detergent reagent was added to the cells. After additional 2 h of incubation in darkness at 37°C, absorbance was read at 570 nm using a spectrophotometer (Multiskan spectrum, Thermo Labsystems).

### Caspase-3 Activity Assay

The caspase-3 activity assay was following the manufacturer’s instructions (Molecular Probes). The EnzChek^§^ caspase-3 assay kit #2 was utilized. In brief, 2 × 10^6^ cells were seeded on the 6-well plates. After the treatment using 500 μM H_2_O_2_ for 1 h, cells were washed with phosphate-buffered saline (PBS) and lyzed using the lysis buffer provided by the manufacturer, followed by centrifugation. Supernatant was transferred to a new white color 96-well plate and added with working solution containing caspase-3 substrate. After covering the plate in dark for 30 min, fluorescence was read with the excitation/emission of ∼496/520 nm on a microplate fluorometer (Fluoscan Ascent FL, Thermo Labsystems).

### Western Blotting Analysis

Western Blotting (WB) analysis was carried out by the method described previously (Hiltunen et al., 2006; Zhang et al., 2007; Choi et al., 2014). Briefly cells were lysed in the radioimmunoprecipitation (RIPA) cell lysis buffer (50 mM Tris-HCL pH 7.4, 150 mM NaCl, 1 mM EDTA, 1% NP-40, 1 mM PMSF and 1 μg/ml aprotinin, 1 μg/ml leupeptin, and 1 μg/ml pepstatin). After centrifugation and protein concentration measurement, equal amount of protein was applied to the electrophoresis followed by membrane transfer, antibody incubation and signal development.

### Aβ Measurement

Aβ measurement was following the method described previously (Hiltunen et al., 2006). In brief, Aβ40 and Aβ42 levels (pg/ml) were quantified using the sandwich enzyme-linked immunosorbent (ELISA) assay. Each experiment was carried out at least in triplicate.

### Statistical Analysis

Values in the text and figures were demonstrated as means ± standard errors from at least three independent experiments. Equal variance two-sample student’s *t*-test was utilized to compare two groups followed by Bonferroni’s test if multiple comparisons were examined within a single experiment.

## Results

### Loss-of-Function in *Drosophila ubqn* Suppresses Human Amyloid-β Protein Precursor and Human BACE Phenotypes in Wing Veins

**FIGURE 1:**
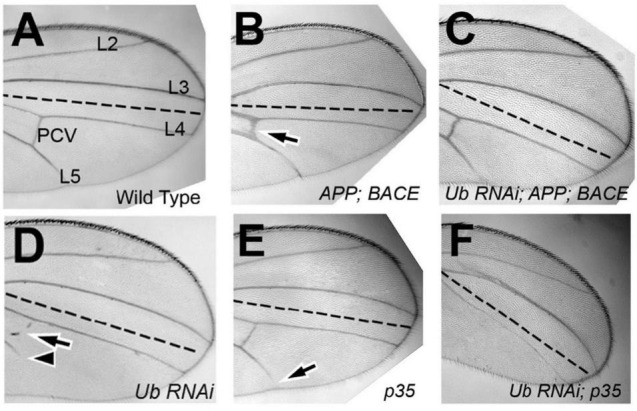
*Drosophila ubqn* genetically interacts with human APP and BACE in the wing. All wings are anterior up, distal right, same magnification. All wings express UAS constructs in the posterior compartment only (as driven by *engrailed:Gal4*). UAS constructs are listed in the bottom right of each panel. Posterior compartments are depicted below the dotted line in each panel. **(A)** Representation of a wild type wing where *UAS:GFP* is expressed. Longitudinal wing veins L2-L5 are depicted, as is the posterior crossvein (PCV). **(B)** Representation of a wing where human APP and human BACE are co-expressed. Arrow shows extra vein and thickening of the PCV. **(C)** Representation of a wing where *ubiquilin-1 RNAi (shUbqln1)* is expressed with human APP and human BACE. Note the suppression of the extra/thick PCV. **(D)** Representation of a wing where *ubiquilin-1 RNAi* is expressed. Arrowhead shows loss of vein L5 material. Arrow shows loss of PCV. **(E)** Representation of a wing where *UAS: p35* is expressed. Arrow shows loss of vein L5. **(F)** Representation of a wing where both *ubiquilin-1 RNAi* and *p35* are co-expressed. Note the enhancement of vein loss in both the PCV and L5 tissue. Ub, Ubiquilin-1.

To analyze the function of *ubqn in vivo*, we used a publicly available *ubqn* RNAi construct (*Ub-RNAi*) under the transcriptional control of the Gal4/UAS system (Brand and Perrimon, 1993). Previous studies have shown that RNAi knockdown of *ubqn* in the entire *Drosophila* wing leads to a strong loss of vein phenotype (Li et al., 2007; Ganguly et al., 2008). To extend these findings, we expressed the well-established and previously reported engrailed-Gal4 which is restricted to the posterior wing compartment (Brower, 1986; Marenda et al., 2006; Vrailas-Mortimer et al., 2007). We analyzed the posterior domain of developing wings (below the dotted line in Figure 1A), and found a strong loss of vein phenotypes, including loss of wing vein L5 in all wings (arrowhead Figure 1D and Table 1), and loss of the posterior cross vein in 91% of wings (arrow in Figure 1D and Table 1). Our findings were consistent with expressing RNAi knockdown of *ubqn* in the entire *Drosophila* wing (Li et al., 2007; Ganguly et al., 2008). These results suggested that our *Ub-RNAi* construct was silencing *ubqn* function through RNAi knockdown.

**TABLE 1:** Genetic Interaction between Ubiquilin, APP, BACE, and p35.

			% showing phenotype					
Gene(s)	Genotype	n	Thick PCV	Extra ACV	Blisters	Loss of L5	Loss of PCV	Nature of Allele
GFP (Control)	en:GFP	100	–	–	–	–	–	Control
Ubiquilin	en:Ubiquilin RNAi	84	–	–	–	100	91	RNAi knockdown
APP	en:App	130	–	–	–	–	–	Over-expression
APP BACE	en:APP; BACE	100	65	60	13	–	–	Over-expression
Ubiquilin APP	en:Ubiquilin RNAi; APP	68	–	–	–	50	95	RNAi knockdown w/overexpression
Ubiquilin APP BACE	en:Ubiquilin RNAi; APP; BACE	106	–	–	2	70	19	RNAi knockdown w/overexpression
p35	en:p35	126	–	–	–	17	11	Over-expression
Ubiquilin p35**	en:Ubiquilin RNAi; p35	59	–	–	53	100	100	RNAi knockdown w/overexpression

**en:Ubiquilin RNAi; p35 wings also displayed 17% of wings with loss of wing vein L4 in addition to vein L5.

*PCV, Posterior crossvein; ACV, Anterior crossvein.*

While expression of human APP in the posterior compartment of wings had no effect on adult wing vein structures (Table 1), expression of human APP with human BACE led to extra vein phenotypes (Figure 1B), with thicker posterior crossveins, extra anterior crossveins, and blistering (Table 1). This is consistent with what has been previously reported by Greeve et al. (2004). Specifically, Greeve et al. showed that expression of both BACE and Presenilin had no extra vein phenotype in wings, nor did expression of APP alone (Fossgreen et al., 1998). Taken together with our results, we suggest that this indicates the extra vein phenotypes observed are due to human β-secretase cleavage of expressed human APP protein. It is also important to note that previous analysis has shown that replacing human APP with *Drosophila* APPL protein, which does not contain an Aβ domain, suppressed extra vein formation, indicating the mechanism of extra vein formation is related to the presence of Aβ (Greeve et al., 2004; Li et al., 2007).

When both human APP, human BACE, and *Ub-RNAi* were expressed simultaneously, a strong suppression of both phenotypes associated with *ubqn* knockdown, and phenotypes associated with human APP and human BACE over-expression were observed (Figure 1C and Table 1). Further, suppression of the loss of L5 vein tissue associated with *ubqn* knockdown was also observed upon expression of human APP alone (Table 1). These data indicate that *Drosophila ubqn* facilitates human APP and human BACE in this vein phenotype in the wing, as these phenotypes are suppressed when *ubqn* is knocked down. Ganguly et al. have shown that *Drosophila ubqn* antagonizes *presenilin* function in the wing (Ganguly et al., 2008). Combined with our data, this suggests that *ubqn* function differs between components of the γ-secretase pathway and the β-secretase pathway during differentiation within this tissue.

### *Drosophila ubqn* Genetically Interacts With p35 in Wing Vein Phenotypes

*Drosophila ubqn* has been previously suggested to be pro-apoptotic when over-expressed in the eye (Ganguly et al., 2008). To determine if the phenotypes we observe from *ubqn* knockdown in the *Drosophila* wing were also partially apoptotic in nature, we simultaneously expressed *Ub-RNAi* and the baculovirus pan-caspase inhibitor p35. Expression of p35 alone (*UAS:p35*) showed weak loss of vein structures (Figure 1E and Table 1), in agreement with previous findings (Marenda et al., 2006). Simultaneous expression of both *Ub-RNAi* and p35, showed a strong enhancement of loss of veins structures (Figure 1F and Table 1), in addition to wing blistering. These data indicate that *ubqn* may play a role in wing vein differentiation as a regulator of apoptosis in the *Drosophila* wing.

### *Drosophila ubqn* Alters Cell Number and Tissue Compartment Size in the Wing

Apoptosis is part of the normal development of many epithelial tissues, including the *Drosophila* wing, and is a significant factor in both the pathology of cancer and neurodegenerative diseases. Increased ubiquilin-1 protein, and decreased phosphorylated ubiquilin-1 have been found to be associated with lung adenocarcinoma (Chen et al., 2007), linking ubiquilin-1 function to cancer. Consistent with our data above, we suggest that in the *Drosophila* wing, *ubqn* appears to function in a pro-apoptotic fashionf. Thus, to test whether the phenotypes we observed in the wing were due to increased cell number or due to a loss of apoptosis upon *ubqn* knockdown, we performed cell count and wing surface area measurements of these wings.

**FIGURE 2:**
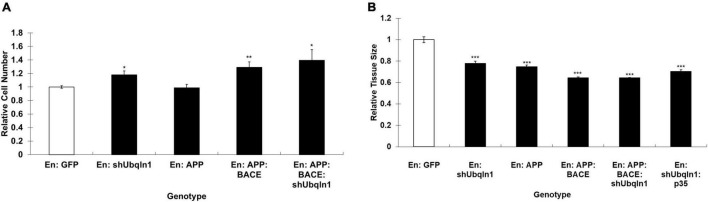
Effects of *Drosophila ubqn* on cell number **(A)** and growth **(B)** in the wing. The *UAS* constructs were expressed in the posterior domain of wings, and analyzed for the difference in cell number between the posterior (P; experimental) and anterior (A; control) compartments. For each genotype, five independent wing compartments were assayed **(A)**. Total cell number for each genotype included: En: GFP (A: 300, 318, 327, 293, 363; P: 480, 566, 542, 499, 569); En: shUbqln1 (A: 275, 342, 318, 351, 317; P: 624, 651, 647, 572, 624); En: APP (A: 380, 355, 305, 270, 335; P: 550, 545, 500, 537, 532); En: APP:BACE (A: 256, 310, 318, 247, 325; P: 647, 615, 570, 601, 651); and En: APP: BACE: shUbqln1 (A: 344, 320, 238, 372, 350; P: 771, 638, 815, 712, 708). *p < 0.05, **p < 0.01; ***p < 0.001; compared to En:GFP. En, engrailed; P, posterior; A, Anterior.

Each wing cell secretes one wing hair (Meyer et al., 2000), which we used as a proxy for cell number. We expressed our *UAS* constructs in the posterior domain of wings, and analyzed the difference in cell number between the posterior (P; experimental) and anterior (A; control) compartments of each genotype. To test specific expression of the constructs in the posterior compartments, we found that anterior compartments of *Drosophila* wing cell number displayed no significant differences comparing animals expressing different constructs in their posterior domain of wings, as expected (Supplementary Figure 1). Because there was no significant difference in cell number between the genotypes within the anterior compartment of wings, we next studied if there were significant differences between posterior compartments. We normalized each posterior cell count first to the anterior cell count of the same genotype, and then to the control cell count where *UAS:GFP* was expressed. We found that upon *ubqn* knockdown, the number of cells within the posterior compartment significantly increased compared to control wings (Figure 2A). Taken together, this suggests that *ubqn* functions to restrict cell number in the developing wing. Expression of human APP had no significant effect on cell number (Figure 2A), but co-expression of both human APP and human BACE increased cell number significantly (Figure 2A). Expression of *Ub-RNAi* with human APP and human BACE slightly increased cell number, indicating that *ubqn* antagonizes human APP and human BACE function in cell number of the wing.

To analyze cell growth within the wing, we expressed our UAS constructs as above, and measured the surface area of both the anterior compartment (control) and posterior compartment (experimental). As with the wing cell counts, we did not observe a significant difference in the surface area of anterior compartments between different genotypes (data not shown). We then normalized each posterior compartment measure as above. We found that upon *ubqn* knockdown, the size of the posterior compartment of adult wings significantly decreased compared to control wings (Figure 2B). When taken together with our cell count data, this suggests that though there are more cells within the posterior compartment of these wings, these cells are significantly smaller in size than normal. We suggest that this is due to a loss of normal apoptosis due to *ubqn* knockdown, resulting in an abnormally larger number of cells within the posterior compartment of these wings, and abnormal tissue morphology.

Interestingly, though expression of human APP in these wings had no effect on cell number, it did decrease overall tissue size (Figure 2B). This effect was enhanced when both human APP and human BACE were simultaneously expressed in these wings (Figure 2B). However, unlike with our cell number results, simultaneous expression of *Ubiquilin RNAi* with human APP and human BACE did not further enhance this effect (Figure 2B). Simultaneous expression of p35 with Ub-RNAi did show an enhancement of the tissue size defect from Ub-RNAi expression alone. These data were consistent with what we observed between these two genotypes in the differentiation of veins in the wing, and strengthen our conclusion that ubqn is normally pro-apoptotic in the developing Drosophila wing.

### Silencing Ubiquilin-1 Alters Cells Viability and Caspase-3 Activity in Human Cells

Because ubiquilin-1 is indicated to be involved in PQC and we showed ubiquilin-1-related abnormality and apoptosis in *Drosophila*, we expect that silencing ubiquilin-1 may change cell viability and lead cells go through apoptosis. Therefore, we next set out to analyze the effects of silencing ubiquilin-1 on cell viability using human cell models. We utilized our previously reported cell lines with ubiquilin-1 stably knocked down using plasmid-based shRNA constructs which contained a puromycin resistance marker (Zhang et al., 2007). Cells stably expressing shRNA control or ubiquilin-1 were generated by selecting for puromycin resistant clones. The clonal cells with puromycin resistant constructs were selected and maintained in 1 μg/ml puromycin and 200 μg/ml G418 containing medium. The clonal cells were analyzed for ubiquilin-1 knock-down by Western blotting analysis with β-actin used as a loading control (Supplementary Figure 2).

Our control and shUbqln1 stably knockdown SY5Y-APP-Gal4 cells were challenged with 500 μM H_2_O_2_ for 1 h and applied to MTT cell viability analysis. We showed that ubiquilin-1 knockdown cells significantly decreased cell viability compared with control cells (*p* < 0.05).

**FIGURE 3:**
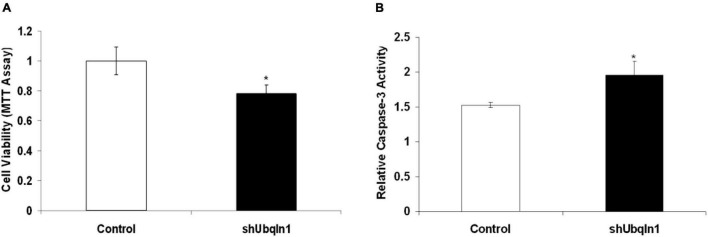
Down-regulation of ubiquilin-1 decreases cell viability and elevates caspase-3 activity. **(A)** Ubiquilin-1 knock-down significantly decreased cell viability in SY5Y-APP-Gal4 cells utilizing the MTT assay (*p* < 0.05). SY5Y-APP-Gal4 cells stably expressing the control (a non-specific shRNA not targeting any known genes) and shUbqln1 were incubated with 500 μM H_2_O_2_ for 1 h, and then subjected to MTT assay to measure cell viability. **(B)** Ubiquilin-1 knock-down significantly elevates caspase-3 activity. SY5Y-APP-Gal4 cells stably expressing the control and shUbqln1 were incubated with 500 μM H_2_O_2_ for 1 h, and then utilized in a caspase-3 activity assay (*p < 0.05). The caspase-3 activity of shUbqln1 cells was compared to control cells.

Because increased caspase-3 activity has been shown to associate with cell viability and Aβ metabolism in AD (Xie et al., 2008), we also analyzed the caspase-3 activity in our cells. We showed that ubiquilin-1 knockdown cells displayed a significant elevation in the caspase-3 activity compared to control cells under the H_2_O_2_ treatment (*p* < 0.05) (Figure 3). These findings support the previous report showing overexpression of *UBQLN1* in association with reduced caspase-3 activity (Liu et al., 2014), as well as a study showing reduced ubiquilin-1 expression in a brain injury mouse model (Luo et al., 2019). Collectively, our cell-based results extend the findings of *Drosophila*, suggesting a loss-of-function mechanism of ubiquilin-1 in AD.

### Ubiquilin-1 Over-Expression Decreases Aβ Levels

**FIGURE 4:**
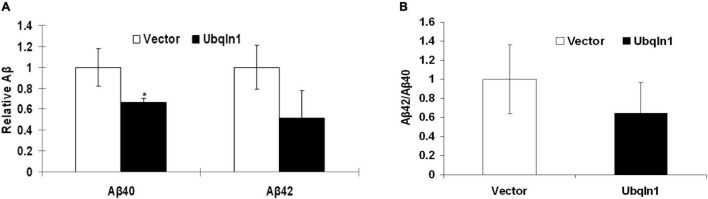
Ubiquilin-1 overexpression decreases Aβ levels. The effects of ubiquilin-1 over-expression on Aβ levels were evaluated using naïve HEK293 cells which were transiently transfected with the ubiquilin-1 or empty vector plasmids. Conditioned media was prepared 48 h post transfection and utilized for Aβ assay by ELISA. **(A)** Ubiquilin-1 over-expression significantly decreased Aβ40 (*p < 0.05) and showed a trend toward decreasing Aβ42 levels and Aβ(42:40) ratios (*p* > 0.05). **(B)** Ubiquilin-1 did not significantly change Aβ(42:40) ratios (*p* > 0.05).

Evidence showed that *UBQLN1* knockdown increases levels of Aβ40 and Aβ42 levels in cell-based models (Hiltunen et al., 2006). However, it hasn’t been demonstrated that increasing ubiquilin-1 expression alone leads to decreased Aβ levels in cell models. To test the effects of increasing ubiquilin-1 on Aβ levels, naïve HEK-293 cells were utilized and transiently transfected with previously reported control or *UBQLN1* plasmid for 48 hrs. Conditioned media was prepared and utilized for the Aβ analysis by ELISA. Overexpression of ubiquilin-1 decreased Aβ40 levels by 40% compared to control (*p* < 0.05) and lowered Aβ42 levels by 60% (*p* = 0.09) (Figure 4A). Increasing ubiquilin-1 reduced Aβ(42:40) ratios by 40%, which however did not reach the statistical significance level (Figure 4B). Collectively, increasing *UBQLN1* reduces Aβ levels, which is associated with the effects of *UBQLN1* deficiency on increasing Aβ levels through a mechanism related to regulation of APP trafficking and metabolism (Hiltunen et al., 2006; Gross et al., 2008). Because reducing Aβ level is a therapeutic strategy for AD treatment, our results as well as others suggest that ubiquilin-1 is an AD therapeutic target and that overexpressing *UBQLN1* may be considered as a potential therapeutic strategy.

### γ-Secretase Inhibition Increases Ubiquilin-1 Protein Levels

**FIGURE 5:**
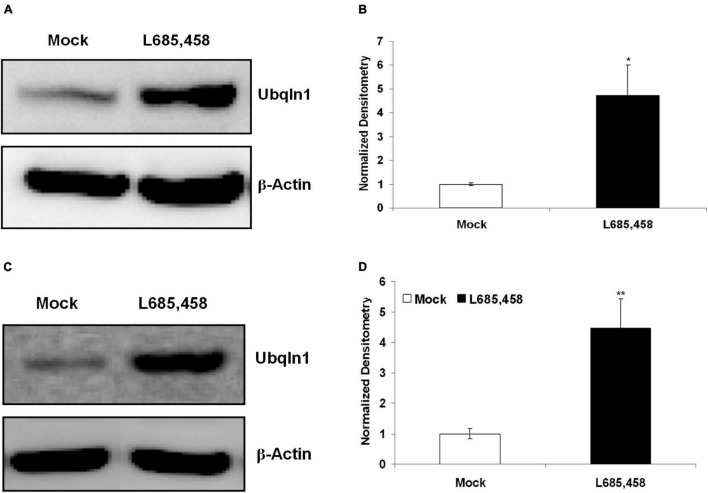
Inhibition of γ-secretase increases ubiquilin-1 protein levels. **(A)** L685,458 increased ubiquilin-1 protein levels in SH-SY5Y cells. Naïve SH-SY5Y cells were treated with the mock solution (DMSO) or 5 μM L685,458 (in DMSO) for 1 h. Cell lysates were prepared and applied to WB analysis. β-Actin served as the loading control. **(B)** Normalized densitometry for **(A)**. *p < 0.05. **(C)** L685,458 increases ubiquilin-1 protein levels in HEK-293 cells. Similar to **(A)**, naïve HEK293 cells were treated with the mock solution or 5μM L685,458 for 1 h. Cell lysates were prepared and applied to WB analysis. β-Actin served as the loading control. **(D)** Normalized densitometry for **(C)**. **p < 0.01.

Previous findings from our group and other groups showed that ubiquilin-1 modulates PS1 proteolysis and γ-secretase activity (Mah et al., 2000; Zhang et al., 2007). We next investigated if regulating γ-secretase activity may change ubiquilin-1 levels. First, we utilized L685,458, a common γ-secretase transition state inhibitor and contained in DMSO (Zhang et al., 2007), on naïve SH-SY5Y cells and cell lysates were then prepared and analyzed by WB. We showed that 5 μM L685,458 robustly increased ubiquilin-1 protein levels up to 5-fold comparing to the mock (DMSO) group (*p* < 0.05) in naïve SH-SY5Y cells (Figures 5A,B). We next attempted to generalize this finding using a different cell model. We utilized our naïve HEK-293 cells and found that L685,458 increased ubiquilin-1 levels up to 4-fold comparing to the mock group (*p* < 0.01) (Figures 5C,D). Thus, our results using pharmacological inhibition of γ-secretase resulted in a strong increase in ubiquilin-1 levels. These findings extends the previous studies showing the protein interactions of ubiquilin-1 and PS1 (Mah et al., 2000) as well as those showing protein-level changes of ubiquilin-1 in various pathophysiological conditions, including AD (Stieren et al., 2011; Luo et al., 2019).

## Discussion

In this study, we investigated the association of ubiquilin-1 with AD by analyzing the effects of ubiquilin-1 in cell and animal models. Particularly, we utilized both cell and animal-based models to analyze the consequences of changing ubiquilin-1 levels on PQC-related events as well as in the interaction of BACE and γ-secretase. We showed that loss of *Drosophila ubiquilin-1* (*ubqn*) function in the wing leads to wing vein phenotypes, altered cell number, and altered tissue compartment size. Our results in the fly suggested that *ubqn* is normally pro-apoptotic in the developing wing, and also functionally interacts with human β-secretase and APP. These findings agreed with AD-related reduction of ubiquilin-1 and decreased quality control of APP (Stieren et al., 2011).

A number of previous studies showed that ubiquilin-1 is a PQC protein and can alter cellular toxicity and apoptosis (Ko et al., 2002; Wang et al., 2006; Zhang et al., 2020). Particularly, apoptosis, or programmed cell death, plays a critical role in development as well as in AD and other neurodegenerative diseases (Kim et al., 1997). Ubiquilin-1 could alleviate hypoxia-induced apoptosis in SH-SY5Y cells (Ko et al., 2002). Furthermore, ubiquilin-1 is a protein involved in the PQC system, which is a complex cellular mechanism that consists of the endoplasmic reticulum (ER) and UPS and other organelles (Ellgaard and Helenius, 2003; Kostova and Wolf, 2003). The ER is a eukaryotic organelle where protein folding and degradation can occur. The UPS is essential in regulating many cellular processes, including cell cycle, gene expression, and responses to oxidative stress. A recent study showed that overexpression of *UBQLN1* reduces neuropathology in a mouse model of amyotrophic lateral sclerosis with frontotemporal dementia (ALS/FTD) that contains a mutation in *UBQLN2*, a gene that encode an ubiquilin-1 homolog protein (Wang et al., 2020). These collective data suggest that ubiquilin-1 is a potential player underlying AD which requires further study.

To analyze the association of ubiquilin-1 with AD, we investigated the effects of ubiquilin-1 on apoptosis and PQC in cell and animal models. We showed that ubiquilin-1 is involved in regulating cell viability and apoptosis activity both *in vitro* and *in vivo*. We identified genetic interactions between human APP, BACE, and *ubqn* in *Drosophila*, which may be due to the effects of these proteins on apoptosis. *Drosophila ubqn* facilitated human APP and human BACE in vein phenotype in the wing when these phenotypes were suppressed with *ubqn* deficiency. Our results of *Drosophila ubqn* in the wing agreed with previous results showing that *Drosophila ubqn* displays pro-apoptotic functions during over-expression in the eye (Ganguly et al., 2008). To gain insight into the regulatory effects of ubiquilin-1 on cell viability, we found that *UBQLN1* knock-down decreased cell viability with elevated caspase-3 activity in the presence of H_2_O_2_ in SH-SY5Y cells. Our finding of ubiquilin-1 in association with apoptosis was supported by previous studies that analyzed ubiquilin-1 in apoptosis (Ko et al., 2002; Liu et al., 2014; Luo et al., 2019; Zhang et al., 2020). We note that apoptosis plays complex roles in both development and neurodegenerative conditions and ubiquilin-1 may modulate apoptosis in different conditions of related tissues (Ko et al., 2002; Ganguly et al., 2008).

**FIGURE 6:**
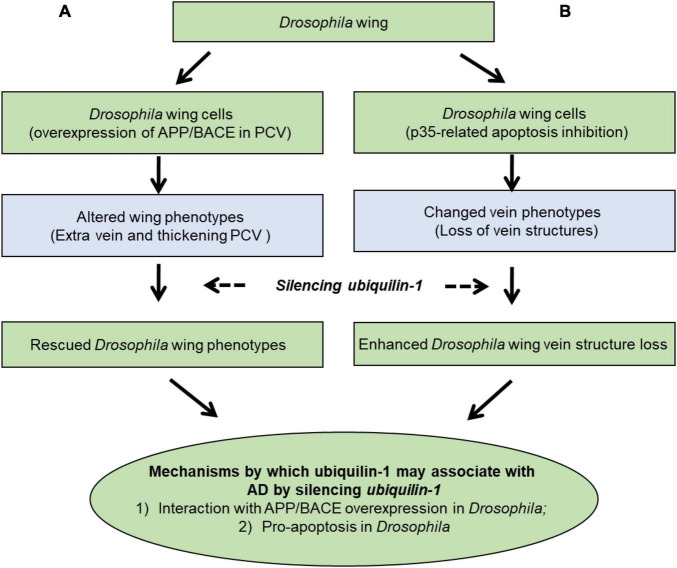
A working model showing mechanisms by which ubiquilin-1 may associate with AD. We characterized the mechanisms by which ubiquilin-1 may associate with AD using *Drosophila* and cell-based models. Our results extended previous reports and suggested two main mechanisms: **(A)** an APP/BACE-related (amyloid-dependent) mechanism, in which APP and BACE overexpression-related altered cell number and tissue compartment size in the *Drosophila* wing posterior crossvein (PCV) is observed and can be rescued by *ubqn* RNAi (left column). **(B)** An apoptosis-related (amyloid-independent) mechanism, in which expression of the baculovirus pan-caspase inhibitor p35 in the *Drosophila* wing resulted in weak loss of vein structures and can be enhanced by *ubqn* RNAi (right column).

Our results support a working model (Figure 6), which demonstrates two main mechanisms by which ubiquilin-1 may associate with AD. First our model showed APP and BACE overexpression-related wing veins and altered cell number and tissue compartment size in the *Drosophila* wing, which can be rescued by *ubqn* RNAi (Figure 6A, left column). This mechanism is likely due to Aβ, which agreed with previous findings of *ubqn* in *Drosophila* (Greeve et al., 2004; Li et al., 2007). The relationship of ubiquilin-1 and Aβ was further supported by our cell-based study overexpressing ubiquilin-1 in human cell models in this study. Next, we expressed the baculovirus pan-caspase inhibitor p35 in the *Drosophila* wing and found weak loss of vein structures, which can be enhanced by *ubqn* RNAi (Figure 6B; right column). This suggested an apoptosis-related (amyloid-independent) mechanism of *Drosophila ubqn, consistent with* a previous study (Ganguly et al., 2008). Additionally, our findings in *Drosophila* were further supported by our results showing an increase of caspase-3 activation caused by H_2_O_2_ *in vitro*. Collectively, our findings further extended previous results, and support an APP/BACE-related (amyloid-dependent) mechanism (Figure 6A) and an apoptosis-related (amyloid-independent) mechanism (Figure 6B) of ubiquilin-1.

Moreover, a number of proteins and pathways have been discovered to be pivotal mediators in apoptosis, e.g., members of the interleukin-1β-converting enzyme (ICE)/Ced-3 proteases (caspases) family of protease (Schlegel et al., 1995, 1996). A hallmark of apoptosis is caspase-mediated cleavage of specific Asp-Glu-Val-Asp (DEVD) amino acid sequence-containing proteins, including poly (ADP-ribose) polymerase (PARP), which is essentially involved in DNA repair in response to environmental and intracellular stress. PARP is a key player for AD pathogenesis and intervention (Salech et al., 2020). Thus, our findings may suggest future studies on ubiquilin-1-related changes in PARP metabolism as well as other AD-related apoptosis and other neuropathology.

Furthermore, our findings support that ubiquilin-1 may be a therapeutic target for the intervention of AD. On the pathophysiological level, ubiquilin-1 is a molecule that regulates PQC and the protein level of ubiquilin-1 reduces in association of AD progression (Stieren et al., 2011). On the therapeutic effects in preclinical models, we showed that overexpression of *UBQLN1* leads to lowered Aβ levels in cells. Furthermore, increasing *UBQLN1* reduces neuropathology in a mouse model of ALS/FTD conferred by the loss of a protein homologous to ubiquilin-1 (Wang et al., 2020).

Interestingly, our current study discovers a new mechanism in which ubiquilin-1 levels are positively regulated by the inhibition of γ-secretase in our cell models. Our results of the effects of L685, 458 may provide insights on the interaction of ubiquilin-1 and PS1 as well as targeting PS1 to influence ubiquilin-1 levels therapeutically. First, the mechanism that inhibition of γ-secretase increases ubiquilin-1 levels may associate with previous studies reporting ubiquilin-1 as a protein that binds PS1 and regulates PS1 endoproteolysis (Mah et al., 2000). Our results suggest a regulatory circuit which functionally coordinates the levels and activities of γ-secretase and ubiquilin-1. Furthermore, because it is important to maintain levels of ubiquilin-1 that is involved in PQC in responses of apoptotic signal and other stresses, our study implicates that targeting γ-secretase may regulate ubiquilin-1 levels in AD. Different from GSI, γ-secretase modulators (GSMs) have been developed that specifically modulate γ-secretase-mediate APP processing and preferentially reduce aggregation-prone Aβ42 other than Aβ40 (Raven et al., 2017; Ward et al., 2017; Rynearson et al., 2021). Future studies will be required to elucidate the mechanisms related to regulation of γ-secretase and changes of ubiquilin-1 levels.

Finally, we note that although our current study has not investigated specific variants of *UBQLN1* and functional domains of ubiquilin-1, it is important that future studies may be performed to identify genetic variants of *UBQLN1* and analyze their effects on PQC and in AD. Particularly, *UBQLN1* is located on chromosome 9q22, and it can encode two isoforms of a protein that contains 589 amino acids (isoform 1) or 561 amino acid (isoform 2). Ubiquilin-1 contains a UBL (ubiquitin-like) domain and a UBA (ubiquitin-associated) domain. Utilizing these functional domains, ubiquilin-1 interacts with intracellular or surface proteins and modulates their stability and/or steady state levels (Mah et al., 2000; Bedford et al., 2001; Ko et al., 2002, 2004; Wu et al., 2002; N’Diaye and Brown, 2003; Ficklin et al., 2005; Regan-Klapisz et al., 2005; Heir et al., 2006). Ubiquilin-1 appears to be an adaptor protein, which binds polyubiquitinated proteins with its UBL or UBA domain and transport them to the proteasome for degradation, thus preventing aggregates of misfolded protein in the cytoplasm (Kleijnen et al., 2000; Funakoshi et al., 2002; Medicherla et al., 2004). Our current study, in combination with these previous findings, collectively warrant further studies to analyze the impacts of genetic variants and functional domains of ubiquilin-1 on cell viability and AD pathology.

In summary, we showed that silencing *UBQLN1* reduces cell viability and increases caspase-3 activity and overexpression of *UBQLN1* lowers Aβ levels, supporting a loss of function mechanism of *UBQLN1* in AD. Furthermore, pharmacological inhibition of γ-secretase increases ubiquilin-1 protein levels, suggesting a mechanism that regulates the levels of ubiquilin-1. Collectively, our results suggest not only a loss-of-function mechanism of ubiquilin-1 in association with AD, but also support the significance of targeting ubiquilin-1 and PQC as potential therapeutic targets for AD.

## Data Availability Statement

The original contributions presented in the study are included in the article/Supplementary Material, further inquiries can be directed to the corresponding author/s.

## Author Contributions

AS and DM performed experimental design and data interpretation. CZ and SI performed the experiments and analyzed the results. CZ, SS, and DM performed literature review and prepared the draft of the manuscript. All authors revised and completed the manuscript.

## Author Disclaimer

The views expressed in this manuscript do not necessarily reflect those of the National Science Foundation or the United States Government.

## Conflict of Interest

The authors declare that the research was conducted in the absence of any commercial or financial relationships that could be construed as a potential conflict of interest.

## Publisher’s Note

All claims expressed in this article are solely those of the authors and do not necessarily represent those of their affiliated organizations, or those of the publisher, the editors and the reviewers. Any product that may be evaluated in this article, or claim that may be made by its manufacturer, is not guaranteed or endorsed by the publisher.
