# Supplementary material for: Association of the Protein-Quality-Control Protein Ubiquilin-1 With Alzheimer’s Disease Both in vitro and in vivo
Source: Front Neurosci. 2022 Mar 17;16:821059. doi: 10.3389/fnins.2022.821059 (PMC8992708; doi:10.3389/fnins.2022.821059)
Supplement: Supplementary file 1 [file Data_Sheet_1.pdf]

SUPPLEMENTARY MATERIAL

SUPPLEMENTARY FIGURES and FIGURE LEGENDS

Association of the Protein-Quality-Control Protein Ubiquilin-1 With Alzheimer's Disease Both in vitro and in vivo

Can Zhang, Shivangi M. Inamdar, Swathi Swaminathan, Daniel R. Marenda, and Aleister J. Saunders

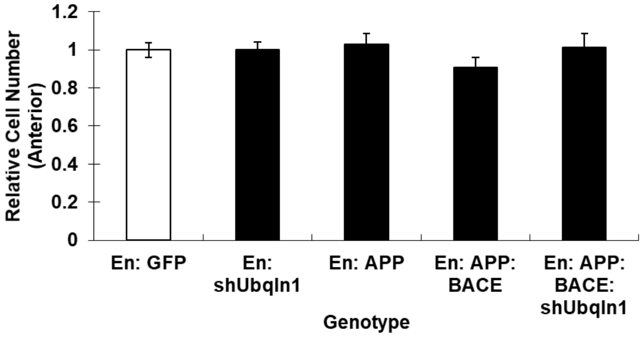

Supplementary Figure 1. **Analysis of anterior compartments of *Drosophila* wing cell number.** *Drosophila* wing cell numbers of the anterior compartments showed no significant differences comparing animals with different *UAS* constructs expressed in their posterior domain of wings. $p > 0.05$ (compared to En:GFP). Abbreviations: En=engrailed.

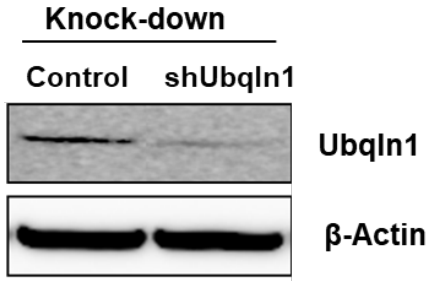

Supplementary Figure 2: Analysis of ubiquilin-1 knockdown in cells expressing shUbqln1 or control shRNA. SY5Y-APP-Gal4 cells were transfected with control shRNA with scrambled sequence or shUbqln1 and selected with 2 µg/ml puromycin for 7 days. Then the clonal cells of puromycin-resistant constructs were selected and analyzed for ubiquilin-1 levels by Western blotting analysis. β-Actin served as a loading control.
